# Hematological parameters and selected intestinal microbiota populations in the Indonesian indigenous crossbred chickens fed basal diet supplemented with multi-strain probiotic preparation in combination with vitamins and minerals

**DOI:** 10.14202/vetworld.2018.874-882

**Published:** 2018-06-29

**Authors:** Sugiharto Sugiharto, Turrini Yudiarti, Isroli Isroli, Endang Widiastuti, Hanny I. Wahyuni

**Affiliations:** Department of Animal Science, Faculty of Animal and Agricultural Sciences, Diponegoro University, Semarang, Central Java, Indonesia

**Keywords:** growth performance, Indonesian indigenous crossbred chickens, intestinal bacteria, minerals, multi-strain probiotic, vitamins

## Abstract

**Aim:**

The aim of this study was to investigate the effect of dietary supplementation with multi-strain probiotic preparation in combination with vitamins and minerals on the hematological parameters and selected intestinal microbiota populations in the Indonesian indigenous crossbred chickens.

**Materials and Methods:**

A total of 240 one-day-old Indonesian indigenous crossbred chicks were raised for 10 weeks. The chicks were distributed to one of four groups, i.e., chicks receiving basal diet without any additive (CONT), chicks receiving basal diet with 0.04% of zinc bacitracin (AGP), chicks receiving basal diet with 0.01% of commercial probiotic *Bacillus subtilis* preparation (PROB1), and chicks receiving basal diet with 0.5% of multi-strain probiotic preparation in combination with vitamins and minerals (PROB2). Blood was collected on the week 8, while the internal organs and eviscerated carcasses were collected on the week 10.

**Results:**

PROB2 tended (p=0.09) to have a lower body weight (BW) compared to CONT chicks. Feed conversion ratio was higher (p<0.05) in PROB1 and PROB2 compared to CONT birds. The number of thrombocytes tended (p=0.09) to be higher in CONT than in other groups. Antibody titer against Newcastle disease virus vaccine was higher (p<0.05) in PROB1 and PROB2 than in CONT group. Serum triglyceride concentration was lower (p<0.05) in PROB2 than in other birds. AGP chicks had lower (p<0.05) serum total protein and globulin concentrations than CONT and PROB1 chicks. Serum albumin level was lower (p<0.05) in PROB2 than in CONT and PROB1 birds. Albumin to globulin ratio tended (p=0.06) to be higher in AGP than in other birds. Lactose-negative Enterobacteriaceae tended (p=0.07) to be lower in PROB1 and PROB2 than in CONT group. PROB1 and PROB2 tended (p=0.06) to have greater lactic acid bacteria (LAB) population than in CONT and AGP birds.

**Conclusion:**

Multi-strain probiotic preparation in combination with vitamins and minerals was able to improve immune response and control the potentially pathogenic bacteria. However, the additive could not improve the growth performance of the Indonesian indigenous crossbred chickens.

## Introduction

The Indonesian indigenous crossbred chicken is the crossbred offspring of male Indonesian indigenous chicken and female modern chicken (i.e., Isa Brown hen) [1]. The chickens have the exterior appearance and meat characteristics similar to indigenous chickens, but they can reach marketing weight at an earlier age than indigenous chickens [2,3]. With intensive rearing system, the Indonesian indigenous crossbred chickens can reach 850 g at 60-70 days, while indigenous chickens can only reach 500 g at the same age [1,4]. In-feed antibiotics have previously been used to optimize the growth potential and maintain the health status of the Indonesian indigenous and indigenous crossbred chickens [5]. The use of such antibiotics in poultry feed may, however, lead to a controversy related to antibiotic resistance in bacteria both in animals and humans as consumers [6]. With the food safety reason, the application of in-feed antibiotics as growth promoters and antimicrobial agents has been banned in many countries including Indonesia.

At present, there is a growing interest in using antibiotic substitutes in poultry nutrition. Among the substitutes, probiotics have gained more attention from poultry nutritionists. When given in adequate numbers, probiotics can exert health benefits on the host by maintaining a microbial balance in the intestine and improving the intestinal functions [6]. Various types of microorganisms are currently used as probiotics, and that lactic acid bacteria (LAB) are the most common type of bacteria used as probiotics for poultry. In addition to the LAB, probiotic containing *Bacillus* spp. has also attracted considerable interest from the poultry nutritionists. In general, *Bacillus* spp. is able to form endospores, which make *Bacillus*-based probiotics not only could survive in the extreme condition in the gastrointestinal tract but also have better viability and stability especially during feed processing and storage [7]. The latter properties make *Bacillus* probiotics seems to be more effective and practical, as compared to LAB-based probiotics, in poultry production.

Under a certain condition such as heat stress, extra supplementations of vitamins, minerals, and probiotics and other additives have commonly been conducted to maintain the health and promote growth rate in broilers fed commercial rations [8]. With regard to probiotics, such additive has often been used in combination with vitamins and minerals to further exert positive impacts on the host. Indeed, daily supplementation of probiotic bacteria plus vitamins and minerals improved immunity and protected the human body from infections, better than placebo [9]. In poultry, we have recently shown that supplementation with probiotic *Bacillus* plus vitamins and minerals (later called multi-strain probiotic preparation in combination with vitamins and minerals) improved physiological conditions and intestinal development of broiler chicks during the brooding period [10]. The treatment also improved digestive functions, physiological status, and an intestinal population of the LAB in broiler chickens [11,12]. The use of such latter additive has, however, never been reported in the Indonesian indigenous crossbred chickens.

The aim of this study was to investigate the effect of dietary supplementation with multi-strain probiotic preparation in combination with vitamins and minerals on the hematological parameters and selected intestinal microbiota populations in the Indonesian indigenous crossbred chickens.

## Materials and Methods

### Ethical approval

The trial was carried out following the standard procedures of rearing and treating of farm animals stated in law of the Republic of Indonesia number 18, 2009, regarding animal husbandry and health.

### Experimental design and management

Two hundred and forty one-day-old Indonesian indigenous crossbred chicks (body weight [BW]=37.8±0.17 g and means±standard deviation) were raised for 10 weeks. In the present study, the chicks were not sexed. Different from broiler chicks, sexing is not commonly done for 1-day-old Indonesian crossbred chicks [13]. At day 0, the chicks were weighed and randomly distributed to one of four experimental groups of 60 chicks each (6 replicates of 10 chicks). The birds were raised in an open-sided house with a wire floor pens throughout the experiment. Light was provided 24 h per day. Plastic curtains, light bulbs (as a heater), and blower fans were installed to control temperature and humidity of the poultry house. The experimental groups included chicks receiving basal diet without any additive (CONT), chicks receiving basal diet with 0.04% of zinc bacitracin (AGP), chicks receiving basal diet with 0.01% of commercial probiotic *Bacillus subtilis* preparation (PROB1), and chicks receiving basal diet with 0.5% of multi-strain probiotic preparation in combination with vitamins and minerals (PROB2). The additives were added at the expense of the basal diets. The diets (in mash form) and water were provided *ad libitum* throughout the study period. The diets were free from coccidiostat, enzymes, and other feed additives/supplements. As there is no standard of nutrient requirement for the Indonesian indigenous crossbred chickens, the basal diet was formulated (Table-1) [12,14] according to the nutrient requirement for broiler chickens (the Indonesian National Standards for Broiler Feed) [15]. The diet contained 21.7% crude protein and 3.286 kcal/kg metabolizable energy (determined according to Bolton [14]). The diet was prepared as a single diet and provided throughout the experiment. The multi-strain probiotic preparation contained 12.10 log cfu/g multi-strains *Bacillus* (i.e., *Bacillus cereus* strain SIIA_Pb_E3, *Bacillus licheniformis* strain FJAT-29133, Bacillus megaterium strain F4-2-27, and Bacillus spp. 11CM31Y12), 0.100 mg Vitamin A, 0.018 mg Vitamin D_3_, 0.100 mg Vitamin E, 1200 mg Ca, 750 mg P, 0.08 mg Mg, 0.006 mg Co, 0.045 mg Cu, 0.015 mg Se, 0.180 mg S, 0.010 mg Zn, 0.060 mg KCl, 0.030 mg I, 0.060 mg Fe, and 0.100 mg Mn [10-12].

**Table-1:** Ingredients and chemical composition (as-dry basis) of basal diet provided to the Indonesian indigenous crossbred chickens^1^.

Items	Composition (%, unless otherwise noted)
Yellow corn	45.5
Soybean meal	17.0
Wheat flour	10.0
Bread flour	5.00
Rice bran	4.45
Crude palm oil	3.50
Corn gluten meal	3.60
Distiller dried grains	3.00
Meat bone meal	2.80
Hydrolyzed chicken feather meal	2.00
Bone meal	1.50
Lysine	0.55
Methionine	0.37
L-threonine	0.08
Salt	0.15
Premi×^2^	0.50
Analyzed composition	
Metabolizable energy (kcal/kg)^3^	3286
Crude protein	21.7
Crude fat	5.90
Crude fiber	6.79
Ash	10.9

1 The basal diet was similar as used in Sugiharto *et al*. [12].

2 Mineral-vitamin premix contained (per kg of diet) of Ca 2.250 g, p 0.625 g, Fe 3.570 mg, Cu 0.640 mg, Mn 5.285 mg, Zn 0.003 mg, Co 0.001 mg, Se 0.013 mg, I 0.016 mg, Vitamin A 375 IU, Vitamin D 150 IU, Vitamin E 0.080 mg.

3 Value was calculated according to formula[14] as follow: 40.81 (0.87 [crude protein+2.25 crude fat + nitrogen free extract] + 2.5). CP=Crude protein

At day 0, commercial Newcastle disease virus (NDV) and avian influenza virus (AIV) vaccines were administrated intramuscularly. Birds were subsequently vaccinated with NDV vaccine through drinking water at week 2 and 5. At the end of the trial, live BW and accumulative feed intake (FI) of chicks were recorded. For complete blood counts and serum biochemical analyses, blood was obtained from the bird’s wing veins (6 birds per treatment) and placed in ethylenediaminetetraacetic acid-containing vacutainers and vacutainers without anticoagulant, respectively, at week 8. Blood in the latter vacutainers was permitted to clot at room temperature and centrifuged at 448 g for 15 min to produce serum. The serum was frozen until analyses [16]. Only male birds from each replicate were used for blood collection. At the end of the study (week 10), the same birds that were blood sampled (6 birds per treatment) were slaughtered, de-feathered, and eviscerated. Immediately, the immune and visceral organs were obtained and weighed [16]. For the bacteriological analysis, ileum (from Meckel’s diverticulum to a point 4 cm proximal to the ileocecal junction) was gently squeezed, and digesta was expelled into the sterile sample bottles. Carcass traits of chicks were determined according to Sugiharto *et al*. [11].

A full blood count was measured using a hematology analyzer (Prima Fully-auto Hematology Analyzer, PT. Prima Alkesindo Nusantara, Jakarta, Indonesia) according to the manufacturer’s instructions. The serum NDV and AIV antibody titers were determined based on hemagglutination inhibition (HI) test [17]. The titers were showed as geometric mean titers (Log_2_). The enzymatic colorimetric/color method was used to measure the levels of total triglyceride, total cholesterol, high-density lipoprotein, and low-density lipoprotein cholesterol in serum. Total protein and albumin in serum were determined according to the spectrophotometric/photometric tests. The difference between total protein and albumin in serum was assigned as the values of globulin. The biochemical analyses in serum were conducted using kits (DiaSys Diagnostic System GmbH, Holzheim, Germany) according to the manufacturer’s instructions. The selected bacterial populations in the ileal digesta of chicks were determined based on Sugiharto *et al*. [18]. Coliform bacteria and lactose-negative Enterobacteriaceae were counted on MacConkey agar (Merck KGaA, Darmstadt, Germany) after aerobic incubation at 38°C for 24 h as red and colorless colonies, respectively. The numbers of coliform and lactose-negative Enterobacteriaceae were assigned as enterobacteria. LAB were counted on de Man, Rogosa and Sharpe (MRS; Merck KGaA, Darmstadt, Germany) agar following anaerobic incubation at 38°C for 48 h.

### Statistical analysis

The data were analyzed based on a completely randomized design by ANOVA using the General Linear Models Procedure in SAS (SAS Inst. Inc., Cary, NC, USA). The pen was regarded as the experimental unit. The significant differences (p<0.05) among experimental groups were further analyzed using Duncan’s Multiple Range Test.

## Results

### Production performances of chicks

The data on final BW, accumulative FI and feed conversion ratio (FCR) are presented in Table-2. The significant difference was not observed in the final BW of crossbred chickens, but there was a tendency (p=0.09) that chicks in the PROB2 group had lower final BW as compared to CONT group. FCR was higher (p<0.05) in PROB1 and PROB2 as compared to CONT, but the difference was not observed between PROB1 and AGP and between PROB1 and PROB2 birds. Accumulative FI was not different among the experimental groups.

**Table-2:** Effect of multi-strain probiotic preparation in combination with vitamins and minerals on performances of the Indonesian indigenous crossbred chickens at 10 weeks of age.

Items	Dietary treatments				SE	p-value

	CONT	AGP	PROB1	PROB2		
Live BW (g)	881	865	838	830	15.1	0.09
Accumulative FI (g)	2639	2646	2734	2737	36.6	0.11
FCR (g/g)	3.13^c^	3.20^bc^	3.42^ab^	3.47^a^	0.07	0.01

CONT = Chicks receiving basal diet without any additive, AGP = Chicks receiving basal diet with 0.04% of zinc bacitracin, PROB1 = Chicks receiving basal diet with 0.01% of commercial probiotic *Bacillus subtilis* preparation, PROB2: Chicks receiving basal diet with 0.5% of multi-strain probiotic preparation in combination with vitamins and minerals, BW = Body weight, FI = Feed intake, FCR = Feed conversion ratio, SE = Standard error

### Internal organ weight of chicks

The data on the internal organs relative weight of the Indonesian indigenous crossbred chickens are presented in Table-3. In general, there was no significant difference in internal organs relative weight among the groups of the indigenous crossbred chickens.

**Table-3:** Effect of multi-strain probiotic preparation in combination with vitamins and minerals on internal organs relative weight of the Indonesian indigenous crossbred chickens.

Items (% live BW)	Dietary treatments				SE	p value

	CONT	AGP	PROB1	PROB2		
Heart	0.73	0.73	0.86	0.66	0.09	0.48
Liver	2.23	2.24	2.45	2.22	0.10	0.37
Proventriculus	0.50	0.53	0.59	0.53	0.03	0.22
Gizzard	1.63	1.60	1.91	1.76	0.12	0.26
Spleen	0.26	0.20	0.26	0.24	0.04	0.63
Thymus	0.54	0.45	0.44	0.53	0.07	0.69
Bursa of Fabricius	0.20	0.13	0.18	0.13	0.04	0.33
Duodenum	0.58	0.72	0.66	0.63	0.05	0.32
Jejunum	1.06	1.19	1.19	1.08	0.09	0.68
Ileum	0.83	0.84	0.78	0.76	0.07	0.83
Caeca	0.48	0.43	0.45	0.44	0.04	0.75
Pancreas	0.27	0.33	0.30	0.29	0.02	0.17

CONT=Chicks receiving basal diet without any additive, AGP=Chicks receiving basal diet with 0.04% of zinc bacitracin, PROB1=Chicks receiving basal diet with 0.01% of commercial probiotic *Bacillus subtilis* preparation, PROB2=Chicks receiving basal diet with 0.5% of multi-strain probiotic preparation in combination with vitamins and minerals, BW=Body weight, SE=Standard error

### Hematological parameters of chicks

The data on complete blood counts of crossbred native chickens are presented in Table-4. The significant difference in full blood counts was not found among treatment groups, but the number of thrombocytes tended (p=0.09) to be higher in CONT than in other dietary treatment groups. The data on antibody titer against NDV and AIV vaccines are shown in Table-5. Antibody titer against NDV was higher (p<0.05) in PROB1 and PROB2 when compared with that in CONT group, but the difference was not significant when compared with that in AGP group. Antibody titer against AIV was not detected in the serum of crossbred chickens during the analysis. The data on serum biochemical parameters of the Indonesian indigenous crossbred chickens are presented in Table-6. Total triglyceride concentration was lower (p<0.05) in the serum of PROB2 than that in other birds. The chicks in AGP group had lower (p<0.05) total protein and globulin concentrations in the serum as compared to CONT and PROB1 chicks, but the difference was not substantial when compared with those in PROB2 chicks. Moreover, serum albumin concentration was lower (p<0.05) in PROB2 than that in CONT and PROB1 birds, but it did not significantly differ from that in AGP birds. Albumin to globulin (A/G) ratio tended (p=0.06) to be higher in AGP as compared to other birds.

**Table-4:** Effect of multi-strain probiotic preparation in combination with vitamins and minerals on complete blood counts of the Indonesian indigenous crossbred chickens.

Items	Dietary treatments				SE	p-value

	CONT	AGP	PROB1	PROB2		
Hemoglobin (g/dL)	10.0	9.12	10.2	9.67	0.50	0.39
Erythrocytes (10^6^/mL)	2.05	1.94	2.07	2.01	0.10	0.76
Hematocrit (%)	28.7	26.4	29.4	27.4	1.41	0.43
Leukocytes (10^3^/mL)	28.0	29.3	27.8	23.1	2.97	0.41
Heterophils (10^3^/mL)	1.84	1.95	1.62	1.18	0.37	0.42
Eosinophils (10^3^/mL)	1.74	1.65	1.76	1.35	0.15	0.18
Lymphocytes (10^3^/mL)	24.4	25.7	24.4	20.5	2.63	0.48
Thrombocytes (10^3^/mL)	18.8	14.3	15.8	14.7	1.33	0.09

CONT=Chicks receiving basal diet without any additive, AGP=Chicks receiving basal diet with 0.04% of zinc bacitracin, PROB1=Chicks receiving basal diet with 0.01% of commercial probiotic *Bacillus subtilis* preparation, PROB2=Chicks receiving basal diet with 0.5% of multi-strain probiotic preparation in combination with vitamins and minerals, SE=Standard error

**Table-5:** Effect of multi-strain probiotic preparation in combination with vitamins and minerals on antibody titer against NDV and AIV of Indonesian indigenous crossbred chickens.

Items (Log_2_GMT)	Dietary treatments				SE	p-value

	CONT	AGP	PROB1	PROB2		
Antibody titer against NDV	2.00^b^	2.83^ab^	3.33^a^	3.00^a^	0.29	0.03
Antibody titer against AIV	ND	ND	ND	ND	-	NA

CONT=Chicks receiving basal diet without any additive, AGP=Chicks receiving basal diet with 0.04% of zinc bacitracin, PROB1=Chicks receiving basal diet with 0.01% of commercial probiotic *Bacillus subtilis* preparation, PROB2=Chicks receiving basal diet with 0.5% of multi-strain probiotic preparation in combination with vitamins and minerals, NDV=Newcastle disease virus, AIV=Avian influenza virus, GMT=Geometric mean titer, SE=Standard error, ND=Not detected (not reached detection level), NA=Not statistically analyzed

**Table-6:** Effect of multi-strain probiotic preparation in combination with vitamins and minerals on serum biochemical parameters of the Indonesian indigenous crossbred chickens.

Items	Dietary treatments				SE	p-value

	CONT	AGP	PROB1	PROB2		
Total cholesterol (mg/dL)	104	109	111	91.9	9.70	0.50
HDL (mg/dL)	58.2	56.0	52.8	63.2	5.19	0.56
LDL (mg/dL)	30.3	35.6	39.8	23.9	9.25	0.62
Total triglyceride (mg/dL)	104^a^	91.1^a^	92.5^a^	57.3^b^	9.55	0.01
Total protein (g/dL)	5.02^a^	3.77^b^	5.03^a^	4.47^ab^	0.30	0.02
Albumin (g/dL)	1.67^a^	1.50^ab^	1.68^a^	1.43^b^	0.06	0.02
Globulin (g/dL)	3.35^a^	2.27^b^	3.35^a^	3.03^ab^	0.29	0.04
A/G ratio	0.51	0.71	0.54	0.49	0.06	0.06

CONT=Chicks receiving basal diet without any additive, AGP=Chicks receiving basal diet with 0.04% of zinc bacitracin, PROB1=Chicks receiving basal diet with 0.01% of commercial probiotic *Bacillus subtilis* preparation, PROB2=Chicks receiving basal diet with 0.5% of multi-strain probiotic preparation in combination with vitamins and minerals, HDL=High-density lipoprotein, LDL=Low-density lipoprotein, A/G ratio=Albumin to globulin ratio

### Bacterial population in the ileum of chicks

The populations of selected bacteria in the ileum of the Indonesian indigenous crossbred chickens are shown in Table-7. Lactose-negative Enterobacteriaceae tended (p=0.07) to be lower in PROB1 and PROB2 groups as compared to CONT group. There was also a tendency (p=0.06) that birds in PROB1 and PROB2 had higher numbers of LAB as compared to CONT and AGP birds. The numbers of enterobacteria and coliform bacteria were not different among the treatment groups.

**Table-7:** Effect of multi-strain probiotic preparation in combination with vitamins and minerals on selected bacterial populations in the ileum of the Indonesian indigenous crossbred chickens.

Items (log cfu/g)	Dietary treatments				SE	p-value

	CONT	AGP	PROB1	PROB2		
Enterobacteria	5.50	5.31	5.16	5.15	0.26	0.75
Coliform bacteria	5.08	4.98	5.12	5.07	0.26	0.98
Lactose-negative enterobacteriaceae	5.11	4.10	3.41	3.56	0.46	0.07
LAB	7.30	7.12	7.75	7.67	0.18	0.06

CONT=Chicks receiving basal diet without any additive, AGP=Chicks receiving basal diet with 0.04% of zinc bacitracin, PROB1=Chicks receiving basal diet with 0.01% of commercial probiotic *Bacillus subtilis* preparation, PROB2=Chicks receiving basal diet with 0.5% of multi-strain probiotic preparation in combination with vitamins and minerals, LAB=Lactic acid bacteria, SE=Standard error

### Carcass traits of chicks

The data on carcass traits of crossbred chickens are presented in Table-8. In general, the significant difference was not observed with regard to the relative weight of eviscerated carcass, breast, thigh, drumstick, wing, and abdominal fat among the chicks.

**Table-8:** Effect of multi-strain probiotic preparation in combination with vitamins and minerals on carcass traits of the Indonesian indigenous crossbred chickens.

Items	Dietary treatments				SE	p-value

	CONT	AGP	PROB1	PROB2		
(% live weight)						
Eviscerated carcass	63.5	65.9	62.0	64.7	1.18	0.14
(% eviscerated carcass)						
Breast	23.5	22.6	23.4	23.7	0.52	0.51
Thigh	15.9	15.3	15.2	15.8	0.25	0.16
Drumstick	15.7	15.4	15.0	15.3	0.33	0.54
Wing	14.1	14.7	14.4	14.2	0.27	0.41
Abdominal fat	0.47	0.53	0.36	0.54	0.21	0.91

CONT=Chicks receiving basal diet without any additive, AGP=Chicks receiving basal diet with 0.04% of zinc bacitracin, PROB1=Chicks receiving basal diet with 0.01% of commercial probiotic *Bacillus subtilis* preparation, PROB2=Chicks receiving basal diet with 0.5% of multi-strain probiotic preparation in combination with vitamins and minerals, SE=Standard error

## Discussion

In the present study, the chicks reached the BW of 854±38.5 g at week 10. This finding was concomitant with Pramono [1] and Widodo *et al*. [4] reporting that the average BW of the Indonesian indigenous crossbred chickens was 850 g at week 8-10. Data in the present study did not show any effect of AGP and probiotic treatments on the final BW of the Indonesian indigenous crossbred chickens. Indeed, FCR was higher in chicks fed a diet containing either commercial *B. subtilis* or multi-strain probiotic preparation in combination with vitamins and minerals, when compared with that in CONT birds. The exact reason for the latter condition remains unclear, but the unbalanced proportion of male and female chicks among treatment groups may be responsible. At the beginning of this study sexing was not conducted and, unfortunately, at the end of the study more male chicks were seen in CONT group than in other treatment groups. Note that male chicks generally have higher and lower final BW and FCR, respectively, when compared with female ones [19]. In Indonesia, the indigenous crossbred chickens are generally produced by the traditional breeders. Unlike modern broiler strains, sexing is not commonly done in the Indonesian indigenous crossbred chickens [13]. The traditional breeders may not be able to employ trained professionals to do vent sexing (accurate sexing method) in chicks. If necessary, the traditional breeders may only perform feather sexing, but the results are less accurate for the indigenous crossbred chicks [13,20]. These aforementioned conditions may explain why sexing is not practicable for 1-day-old Indonesian indigenous crossbred chicks. Referring to the above circumstances and also to several authors who used unsexed chicks during the chicken studies [21-23], we therefore, decided to use unsexed indigenous crossbred chicks during the present trial. In this study, FCR was not different between PROB1 and PROB2 chicks, suggesting that multi-strain probiotic preparation in combination with vitamins and minerals may exert the same effects as commercial *B. subtilis*. However, multi-strain probiotic preparation in combination with vitamins and minerals may not be able to substitute the role of AGP in crossbred chickens, as the FCR was higher in PROB2 than in AGP birds.

There was a tendency that AGP and probiotic treatments resulted in lower values of thrombocytes when compared with control. Ferdous *et al*. [24,25] pointed out that thrombocytes can be an indicator of inflammation (as a result from infections), and that the increased thrombocytes levels may be attributable to infections in the body of animals. In this respect, the lower levels of thrombocytes in the AGP and probiotic treated chicks may suggest the lower infection levels in the respective birds. This inference was supported by another fact in the present study that AGP and probiotic treatments resulted in higher levels of antibody titer against NDV, and thus more protective primarily to ND. Allan and Gough [26] suggested that HI titers of below 3 Log_2_ are regarded as negative for specific immunity against ND, and such condition was found in CONT birds in the present study. The mechanisms through which probiotics increase the antibody titer of chicks were largely unknown, but such microorganisms may modulate the immune system resulting in improved immune responses of birds [6]. Different from antibody titer against NDV, antibody titer against AIV was not detected in the present study. In the earlier study, Rahman *et al*. [27] reported that the HI antibody titers declined with the time postvaccination. In this trial, AI vaccination was conducted on day 0, and therefore the HI antibody titers against AIV may no longer be detected at week 8.

Result in the present study showed that serum total protein and globulin concentrations were lower in AGP when compared with those in other groups. It was apparent in this study that the lower level of total protein in AGP birds was a consequence of the lower globulin in the respective birds. It has been clear from the previous study that the lowered serum globulin concentration was attributed to the damage of hepatocytes in animals [28,29]. With regard particularly to AGP chicks, zinc bacitracin administration seemed to induce dysbiosis (disruption of commensal bacterial community in the intestine) in crossbred chicks [30,31]. The latter condition may damage the intestinal barrier and induce bacterial translocation resulting in liver damage [32]. In this study, AGP birds had higher A/G ratio when compared with other birds. In such case, the relatively lower serum globulin concentration in AGP birds than in other birds may be responsible. In addition to dysbiosis, the lower globulin concentration in AGP birds was also most likely due to coccidian infection in the respective birds. Gao *et al*. [33] reported that coccidian infection was associated with the lowered total protein and globulin levels in the serum of broilers. Similarly, Ola *et al*. [34] showed that coccidian infection resulted in decreased total proteins, albumin, and globulin with an increase of A/G ratio in broiler chickens. In respect to albumin, result in the present study showed that albumin concentration was lowest in PROB2 birds. It has been suggested that higher serum albumin concentration was attributed to the higher BW gain of broiler chickens [35]. Furthermore, Dong *et al*. [36] reported that fat birds had higher serum albumin concentration than lean broilers. In accordance with these earlier reports, we found the lower albumin concentration in PROB2 birds was associated with the lower live BW and higher FCR in the respective birds.

In this study, total triglyceride was lower in the serum of PROB2 compared to that in other birds. This was in accordance with Yang *et al*. [37] reporting a decrease in total plasma triglyceride in broilers treated with *B. subtilis* KT260179. Furthermore, Hatab *et al*. [38] reported that probiotic *B. subtilis* and *Enterococcus faecium* decreased the concentration of triglyceride in the serum of broiler chicks. The present finding may, therefore, indicate that probiotic *Bacillus* was capable of reducing lipid synthesis and/or increasing lipid hydrolysis in the liver [37], resulting in lower concentration of triglyceride in the circulation of the Indonesian indigenous crossbred chickens. However, it is difficult to infer that the decreased serum total triglyceride level in PROB2 birds was simply due to probiotic effect, as the level of total triglyceride in the serum of PROB1 did not differ from that in CONT and AGP groups. It was shown by Hung *et al*. [39] that *Bacillus coagulans* ATCC 7050 had no effect on the serum triglyceride level of broiler chicks, which were concomitant with the absence effect of commercial *B. subtilis* on the serum triglyceride of PROB1 birds. In this respect, certain vitamins and minerals (administrated in combination with probiotics) in PROB2 group may contribute in lowering the level of serum triglyceride of chicks. Our latter inference was supported by Kanchana and Jeyanthi [40] showing the decreased total cholesterol and triglyceride in the plasma of layer chickens with feeding Vitamin E and/or Selenium.

It was shown in the present study that both probiotic treatments (PROB1 and PROB2) decreased the number of lactose-negative Enterobacteriaceae (included in this group among others *Salmonella*, *Proteus*, and *Pseudomonas aeruginosa* and *Shigella*) in the ileal digesta of crossbred chickens. Moreover, the treatment increased the number of LAB in the ileum of chicks. In accordance with our data, Jeong and Kim [41] demonstrated that feeding *B. subtilis* C-1302 decreased and increased the numbers of *Salmonella* and *Lactobacillus*, respectively, in the ileal digesta and excreta of broiler chickens. Hence, feeding *Bacillus* probiotics may produce favorable effects on the intestinal health and reduce the risk of infections in the Indonesian indigenous crossbred chickens. The mode of actions by which *Bacillus* probiotics decreased the counts of lactose-negative Enterobacteriaceae was not exactly known, but *Bacillus* may produce antibiotics, enzymes or both that can kill or inhibit the growth of harmful bacteria [41]. *Bacillus* may also lower the intestinal pH and thus inhibit the proliferation of detrimental bacteria [42]. Unlike the pathogenic bacteria, the intestinal acidification (as a result of *Bacillus* treatment) may favor the growth of LAB in the intestine of chicks [41,42]. The latter condition seemed to explain the increased number of LAB in the intestine of *Bacillus*-treated chicks in the present study.

It was apparent in the present study that dietary treatments did not produce any effect on carcass traits of the Indonesian indigenous crossbred chickens. Concomitant to our finding, treatment with either AGP [12,16], commercial *B. subtilis* [43] or multi-strain probiotic preparation in combination with vitamins and minerals [11,12] had no impact on the carcass traits of broiler chicks. Different from the above-mentioned studies, Shabani *et al*. [44] and Fouad *et al*. [45] showed the potential of probiotics in increasing the carcass percentage of broiler chickens. The different type of probiotics, breed of chickens, and conditions of the trial may be responsible for these different results above.

It seemed in the present study that the role of vitamins and minerals added to probiotic preparation (PROB2) had a minimum effect on the chicks. This inference was supported by the current findings showing a comparable effect of commercial *B. subtilis* and multi-strain probiotic preparation in combination with vitamins and minerals to most of parameters observed in the present study. Supplementation of certain minerals and vitamins into probiotic preparation was actually intended to compensate the possible damage of vitamins and minerals in the feed due to improper feed handling during transportation and storage [11]. Furthermore, supplementation was subjected to alleviate the detrimental effects of unfavorable conditions such as heat stress on poultry [8]. Indeed, Tirawattanawanich *et al*. [46] revealed that Thai indigenous crossbred chickens had a better tolerance to heat stress than modern broiler chickens. Due to the latter study and the fact that the experiment was conducted during the rainy season (average temperature during the day was 30°C±2°C), the vitamins and minerals supplementation seemed to produce minimal effect as the Indonesian indigenous crossbred chickens less suffered from heat stress.

## Conclusion

It could be concluded from the present study that multi-strain probiotic preparation in combination with vitamins and minerals was able to improve immune response and control the potentially pathogenic bacteria. However, the additive failed to improve the growth performance of the Indonesian indigenous crossbred chickens.

## Authors’ Contributions

SS designed, performed the work and wrote the manuscript, TY, EW, and HIW performed the work and revised the manuscript and II performed the data analysis.
